# Scanning 3DXRD Measurement of Grain Growth, Stress, and Formation of Cu_6_Sn_5_ around a Tin Whisker during Heat Treatment

**DOI:** 10.3390/ma12030446

**Published:** 2019-01-31

**Authors:** Johan Hektor, Stephen A. Hall, N. Axel Henningsson, Jonas Engqvist, Matti Ristinmaa, Filip Lenrick, Jonathan P. Wright

**Affiliations:** 1 Division of Solid Mechanics, Lund University, Box 118, 221 00 Lund, Sweden; nilsaxelhenningsson@gmail.com (N.A.H.); jonas.engqvist@solid.lth.se (J.E.); matti.ristinmaa@solid.lth.se (M.R.); 2 Production and Materials Engineering, Lund University, Box 118, 221 00 Lund, Sweden; filip.lenrick@iprod.lth.se; 3 European Synchrotron Radiation Facility (ESRF), 71 Avenue des Martyrs, 38000 Grenoble, France; wright@esrf.fr

**Keywords:** tin whiskers, scanning 3DXRD, stress, Cu_6_Sn_5_

## Abstract

The 3D microstructure around a tin whisker, and its evolution during heat treatment were studied using scanning 3DXRD. The shape of each grain in the sample was reconstructed using a filtered-back-projection algorithm. The local lattice parameters and grain orientations could then be refined, using forward modelling of the diffraction data, with a spatial resolution of 250 nm. It was found that the tin coating had a texture where grains were oriented such that their *c*-axes were predominantly parallel to the sample surface. Grains with other orientations were consumed by grain growth during the heat treatment. Most of the grain boundaries were found to have misorientations larger than 15${}^{\circ }$, and many coincidence site lattice (CSL) or other types of low-energy grain boundaries were identified. None of the grains with CSL grain boundaries were consumed by grain growth. During the heat treatment, growth of preexisting Cu_6_Sn_5_ occurred; these grains were indexed as a hexagonal η phase, which is usually documented to be stable only at temperatures exceeding 186 ${}^{\circ }C$. This indicates that the η phase can exist in a metastable state for long periods. The tin coating was found to be under compressive hydrostatic stress, with a negative gradient in hydrostatic stress extending outwards from the root of the whisker. Negative stress gradients are generally believed to play an essential role in providing the driving force for diffusion of material to the whisker root.

## 1. Introduction

Coating electronic components with a layer of tin (Sn) is an extensively used method to protect them from corrosion. The low melting point of tin ( 232 ${}^{\circ }C$) also makes it attractive as a material for solders, both in its pure form and alloyed with, e.g., copper or silver. Historically, the most common solders consisted of tin alloyed with lead. Due to the toxicity of lead, the European Union enacted the Restriction of Hazardous Substances (RoHS) act in 2006. This legislation aims to eliminate the use of lead in consumer electronics. Since then, similar legislation has been introduced in countries such as China, South Korea and the USA [1]. The elimination of lead from solders has resulted in the reappearance of an old problem, namely the formation of conducting tin whiskers. Tin whiskers are filamentary grains that spontaneously grow out from the tin surface. Whiskers are known to cause short-circuits and catastrophic failures of electronic components [2]. The first observations of tin whiskers were published as early as in 1951 [3], and shortly after, it was found that whisker nucleation could be significantly reduced by co-deposition of lead into the tin coating [4,5]. Due to the RoHS legislation, this solution is no longer available and further research on why whiskers form and how to prevent them is much needed.

The main driving force behind whisker growth is believed to come from compressive hydrostatic stress gradients in the tin coating [6,7,8,9,10]. The most important cause of stresses is the intermetallic compound (IMC) Cu_6_Sn_5_ that forms in the interface between the tin coating, or solder, and the underlying copper substrate. The whisker grows to relax the stresses in the coating; however, IMC formation will continue as long as there are unreacted copper and tin present. This implies that whisker growth can continue for a very long time. The growth of the Cu_6_Sn_5_ phase as a function of ageing time, *t*, can be expressed as

(1) $$W=W_{0}+At^{n},$$

where $W_{0}$ is the initial size, *A* is a constant and the exponent *n* is indicative of the growth mechanism [11]. At elevated temperatures, the growth of Cu_6_Sn_5_ is proportional to $\sqrt{t}$, i.e., n= 0.5 [11,12,13]. This means that at high temperatures the growth of the IMC is driven by bulk diffusion through the IMC layer. At room temperature, however, linear growth has been observed [14,15].

Three-dimensional X-ray diffraction (3DXRD) [16] and the closely related techniques high-energy diffraction microscopy (HEDM) [17] and diffraction contrast tomography (DCT) [18] have been developed and used at synchrotrons for the past 15 years. These techniques aim at mapping out grain orientations, grain positions, and lattice strain in three dimensions for polycrystalline samples. One limitation of these full-field techniques is that variations of the strain field within the mapped grains are not easily measured. Reconstruction of 3D strain maps with a spatial resolution limited by the size of the beam can be achieved using Differential Aperture X-ray Microscopy (DAXM), which uses a polychromatic X-ray beam to probe only the deviatoric part of the strain tensor [19,20]. To remedy these limitations, Hayashi et al. developed a version of 3DXRD that uses a focussed monochromatic X-ray beam that is scanned across the sample [21,22]. Using reconstruction algorithms from tomography, intragranular variations of grain orientation and unit cell parameters can be measured.

We used the scanning 3DXRD technique to characterise the microstructure around a tin whisker in 3D in terms of grain orientation, hydrostatic stress, and presence of Cu_6_Sn_5_. We also investigated the evolution of the microstructure after a heat treatment. The remainder of the paper is structured as follows. In Section 2, the experimental method and some aspects of the data analysis are discussed. The results of the experiment are presented and discussed in Section 3. The paper is concluded in Section 4.

## 2. Method

### 2.1. Sample Description

**Figure 1:**
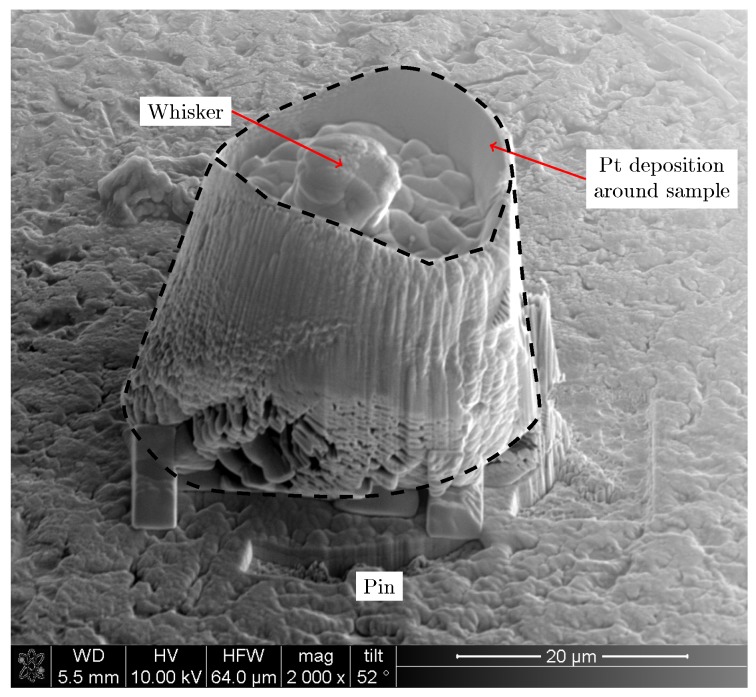
The studied whisker after being lifted out and mounted on a pin using FIB. The sample comprises a 7 μm thick layer of tin deposited on a copper substrate and aged in room temperature for twelve months. The whisker is located in the centre of the sample. The surface of the sample was protected from redeposition during FIB-milling by depositing platinum walls around the whisker. The milled out cylinder was extracted from the substrate using a nanomanipulator and attached to a tungsten pin. The dashed line indicates the perimeter of the extracted cylinder.

For this study, a cylindrical specimen with diameter 25 μm was extracted from a bulk sample by focussed ion beam (FIB) lift-out in an FEI Nova NanoLab 600 microscope equipped with an Omniprobe nanomanipulator. A tin whisker was located in the centre of the cylinder. An SEM image of the specimen is shown in Figure 1. The bulk sample was prepared by coating a high-purity copper substrate with approximately 7 μm of tin using electron beam evaporation. Electron beam evaporation of tin has been shown to produce a columnar grain structure [20], similar to the grain structure obtained by the more industrially relevant method of electroplating [23]. Coatings deposited using electron beam evaporation are also free of organic contaminants which can influence the stress field and the whisker growth kinetics; in electroplated coatings, these contaminants originate from additives in the electrolyte. Prior to the lift-out, the bulk sample was aged in ambient conditions for 12 months.

The lift-out sample was measured using scanning 3DXRD in its aged state as well as after a heat treatment of 150 ${}^{\circ }C$ for three hours. The temperate was chosen to be lower than the phase transformation temperature for the Cu_6_Sn_5_ phase (discussed in Section 3.3) but high enough to give a significant evolution of the microstructure during the short time available for the experiment. The oven was turned off after the heat treatment and the sample was left to cool inside.

### 2.2. Scanning 3DXRD

#### 2.2.1. Data Acquisition

**Figure 2:**
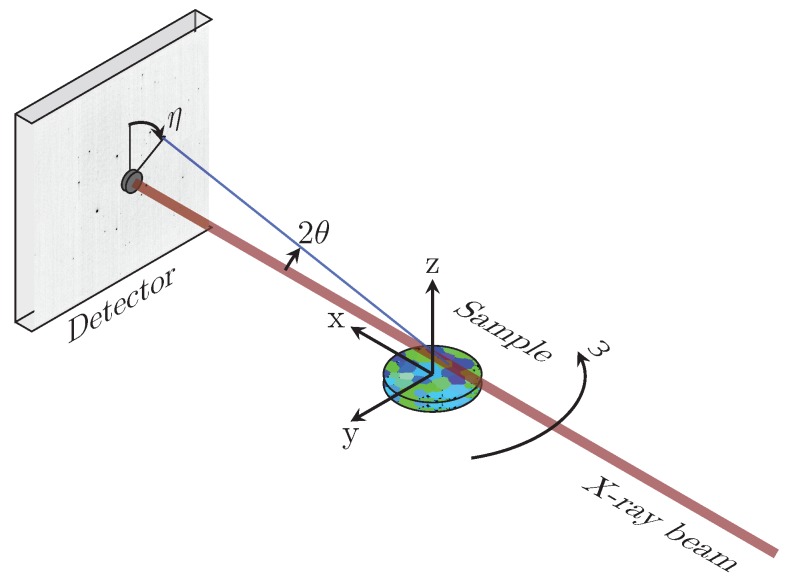
Illustration of the setup for the scanning 3DXRD experiment. The incident beam is focussed to a size smaller than the grain size of the polycrystalline sample, the size of the sample is 25 μm. Diffraction patterns are collected on a 2D detector placed in the far-field regime, 163 mm from the sample. The positions of the diffraction peaks are determined by the angles (2θ,η). The direct beam is absorbed by a beam stop placed in front of the detector. The sample is scanned in the *y* and ω directions, i.e., the rotation axis is scanned across the beam and at each *y* position the sample is scanned in ω. Resolution in three dimensions is obtained by repeating the ω−y scans at several *z*-coordinates.

The synchrotron X-ray experiment was performed using the nanoscope station at the ID11 beamline of the European Synchrotron Radiation Facility (ESRF) in Grenoble, France. A monochromatic beam with an energy of 56.6 keV (wavelength of 0.22 Å) was focussed to a spot size of 250 nm using a pair of silicon compound refractive lenses (CRL) [24]. The size of the beam was significantly smaller than the average grain size of the sample. Diffraction patterns from the volume probed by the X-ray beam were measured on a FReLoN 4M detector with a pixel size of 50 μm located 163 mm behind the sample, using an exposure time of 0.2 s. The cylindrical sample was mounted on an ω rotation stage and *y* and *z* axis translation stages such that the rotation axis for the ω-stage was in the centre of the sample. Figure 2 presents an illustration of the setup and a reference to the coordinate system used. For a given *z*-position (height) in the sample, diffraction patterns were acquired for *y*-motor positions over the range −16 to 16 μm in 250 nm steps. This range was slightly larger than the sample diameter to compensate for the walls of the sample not being completely vertical. For each (y,z) position, ω was scanned from 0 to 180${}^{\circ }$ in 1${}^{\circ }$ steps using an interlaced scanning mode. Such ω−y scans were repeated at a series of different *z*-positions. Results from different *z*-layers were stacked to obtain a 3D volume of rectangular voxels.

The fluorescence signals from the tin coating and the copper substrate were used to monitor the position of the sample and the beam relative to the rotation axis. Based on the fluorescence signal it was possible to compensate for sample drift (due to, e.g., thermal fluctuations). The drift compensation was done halfway through each of the ω scans.

Using the scanning approach described above, each of the ω−y scans took three hours to measure. To make sure that the full depth of the tin coating was scanned, the lowest *z*-coordinate was taken a few micrometres into the copper substrate. The highest *z*-coordinate was located a few micrometres above the sample surface, the full length of the whisker could not be scanned due to time constraints.

#### 2.2.2. Data Analysis

The collected diffraction data were analysed using the Fable software suite [25] and ImageD11 [26]. The background of each pattern was subtracted, and peaks were identified using an intensity threshold of 100 arb.units. The peaks were then indexed and assigned to grains by assigning (*h* *k* *l*) values to two peaks falling on different Debye–Scherrer rings and using these peaks together with an initial guess of the unit cell parameters to refine the orientation and cell parameters of each grain. Only grain orientations that indexed at least 1000 peaks (50 peaks for the IMC grains due to a much lower total number of peaks) were considered. On average, 179,000 diffraction peaks were measured in each of the ω−y scans. The lattice parameters used as initial guesses are presented in Table 1. The result of the indexing was a set of matrices, $\bar{\mathrm{UB}}$, where the columns in each matrix describe the mean reciprocal lattice vectors of one grain [27]. Up until this step, the data analysis is no different than for a standard 3DXRD experiment. To obtain the grain shape and intragranular variations of orientation and unit cell parameters, additional processing of the data was necessary.

**Figure 3:**
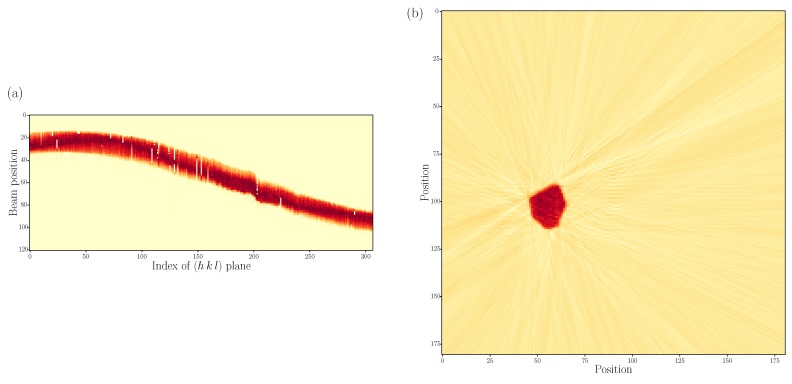
(**a**) Sinogram of one tin grain: The sinogram shows the sum of the intensities of all diffraction peaks belonging to the specific grain as a function of the diffracting lattice planes and the beam coordinate, *y*. The rows of the sinogram are normalised by the maximum intensity at each beam position. (**b**) Grain shape and position for one grain reconstructed by the inverse Radon transform of the sinogram.

The algorithm for refining the intragranular variations and reconstructing the shape of the grains, starting from the average orientation matrices of each grain, is outlined in Algorithm 1. The refinement was performed in two steps, where the first step concerns the reconstruction of the grain shape. For each grain, a sinogram describing the total intensity of the indexed diffraction peaks as a function of *y* and ω coordinates was constructed. The grain shape was reconstructed from the sinograms using filtered back-projection (FBP) with the inverse Radon transform and a ramp filter from scikit-image [28]. One of the sinograms and the reconstruction of the corresponding grain is shown in Figure 3. Voxels were considered to belong to a specific grain if the reconstructed intensity, *I*, was greater than $0.2I_{max}$, where $I_{max}$ is the highest intensity in the FBP reconstruction of that grain.

In the second step of the refinement, the $\bar{\mathrm{UB}}$ matrix was refined locally in each voxel belonging to the current grain. For any given angle, ω, and voxel coordinates, $\left(x_{i},y_{i}\right)$, the *y* scan containing the corresponding data can be found by [21]

(2) $$y\left(\omega \right)=\sqrt{x_{i}^{2}+y_{i}^{2}}\cos\left(\omega +\arctan\frac{x_{i}}{y_{i}}\right).$$

For each voxel, the dataset belonging to the grain being refined and which satisfies Equation (2) was extracted. The extracted dataset was fitted to a forward model of the diffraction to obtain a UB matrix representative of the current voxel. Unfortunately, the extracted dataset not only contains the data from voxel $\left(x_{i},y_{i}\right)$, but is also polluted by data from other voxels illuminated during the same ω−y scans. This pollution of the dataset means that the local refinement is influenced by data from other voxels than $\left(x_{i},y_{i}\right)$. The pollution effect increases with grain size such that all voxels will be reconstructed with the grain average $\bar{\mathrm{UB}}$ matrix for an infinitely large grain.

**Algorithm 1:** Algorithm for reconstruction of intragranular variations of $\bar{\mathrm{UB}}$ matrices.**Input**   **:** Average $\bar{\mathrm{UB}}$ matrices for all grains. Positions of all diffraction peaks. **Output:**
UB matrices, locally refined in each voxel of the grain map. 
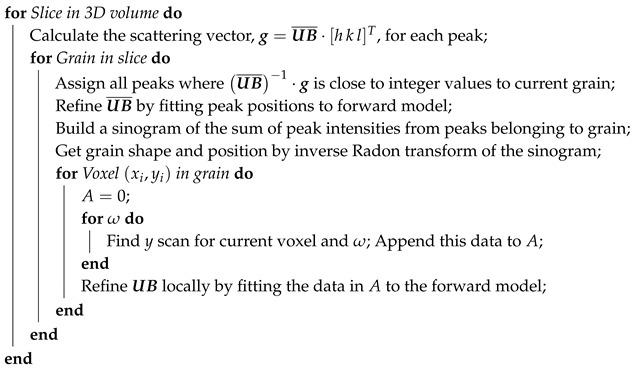


## 3. Results and Discussion

### 3.1. Grain Growth

**Figure 4:**
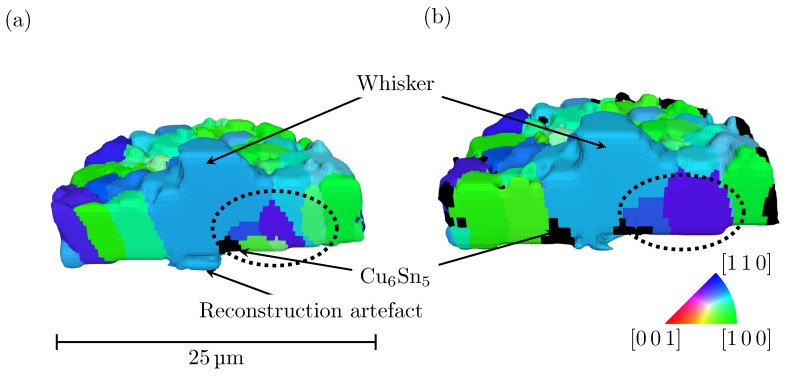
3D reconstruction of the microstructure of the tin coating, the volume is sliced to reveal the columnar structure of the tin grains and the Cu_6_Sn_5_-grains situated below the whisker. The grains are coloured based on the crystal direction parallel to the sample normal: (**a**) before heat treatment; and (**b**) after heat treatment of 150 ${}^{\circ }C$ for three hours.

A 3D reconstruction of the grain structure of the tin coating, before and after heat treatment, is shown in Figure 4. The grains are coloured based on the inverse pole figure of the sample normal, where the Cu_6_Sn_5_ grains are coloured black. It is evident that the coating has a columnar grain structure (one grain throughout the thickness). A similar arrangement was found by Hektor et al. [20]. This type of microstructure is typical for lead-free solders and plays an important role in the formation of whiskers by allowing for fast grain boundary diffusion towards the whisker root [29,30,31,32]. The grain boundaries of the whisker grain can be seen to be tilted with respect to the sample normal. In the whisker growth model proposed by Smetana [33], it is assumed that some recrystallisation mechanism forms grains with oblique grain boundaries; the stress gradient then drives diffusion of atoms towards the newly formed grain, which will grow upwards and become a whisker due to grain boundary sliding. Assuming a biaxial state of stress, the stress in an oblique grain boundary will always be lower in magnitude than if the grain boundary was vertical. It therefore seems reasonable that tin atoms diffuse to grains with these oblique grain boundaries. Stress relaxation in oblique grain boundaries can also explain why very few whiskers form in coatings of lead–tin alloys. These alloys do not have the columnar grain structure that is seen in coatings of pure tin. This allows stress relaxation to take place in the already present oblique grain boundaries, without the need to form a whisker.

Comparing the grain maps before and after the heat treatment, it is seen that coarsening of some of the tin grains took place. One example of this is seen in the region marked by the dotted ellipse in Figure 4. In the original state, this region consists of eight tin grains with different orientations—one of them being the whisker grain—and one grain of Cu_6_Sn_5_. After heat treatment, the same region contains only four tin grains and the grain of Cu_6_Sn_5_. The dark blue and purple grains can be seen to have coarsened and consumed the smaller green grains. The purple grain also grew in the vertical direction and almost reaches the sample surface.

**Figure 5:**
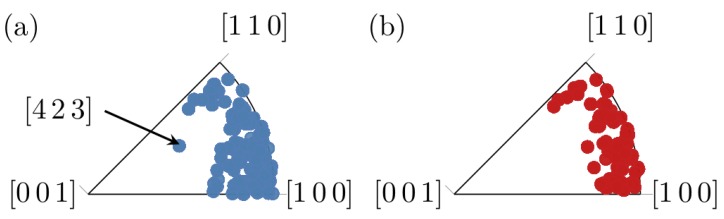
Inverse pole figure showing the crystal orientation parallel to the *z*-axis (sample normal). For reference to the coordinate system see Figure 2. The black arrow marks an orientation which is not present after the heat treatment: (**a**) before heat treatment; and (**b**) after heat treatment.

Figure 5 shows inverse pole figures of the *z*-axis, i.e., the sample normal, for the Sn grains before and after heat treatment. In the original state, most of the grains were oriented in the [1 1 0] or [1 0 0] directions. The effect of the heat treatment is that the few grains with different orientations, such as the [4 2 3] marked in Figure 5, disappeared. This could mean that the grains with orientations other than [1 1 0] or [1 0 0] had higher energy, originating, e.g., from stresses due to the presence of Cu_6_Sn_5_ or from grain boundary misorientation. Therefore, when the mobility of the Sn grains increased during the heat treatment, the grains with high energy get consumed by grains with lower energy.

**Figure 6:**
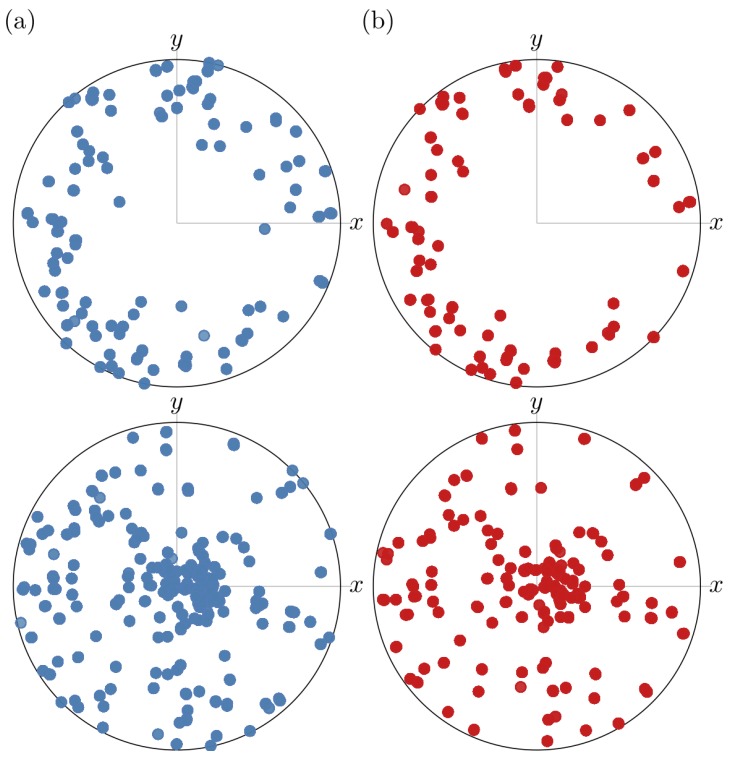
Pole figures showing the direction of the [0 0 1] (top row) and [1 0 0] (bottom row) planes of the Sn crystals. The grains have a [1 0 0] fiber texture where the 〈0 0 1〉 directions lies in the plane parallel to the sample surface: (**a**) before heat treatment; and (**b**) after heat treatment.

The pole figures in Figure 6 show the direction of the normal to the [0 0 1] and the [1 0 0] planes, i.e., the *c*-axis and the *a*- or *b*-axis directions, expressed in the coordinate system shown in Figure 2. These figures confirm that the tin grains had a [1 0 0] fibre texture where the 〈0 0 1〉 directions lie in the plane parallel to the sample surface. The same texture was found on a similar sample by Hektor et al. [10]. The heat treatment did not alter the texture much; there was a small global rotation, which could have been caused by the sample being mounted at a slightly different angle than in the original measurement. The [0 0 1] pole figure after the heat treatment contains fewer poles than before the heating. Most of the poles that disappeared were located in the interior of the pole figure, i.e., they came from grains with orientations deviating from the [1 0 0] fibre texture.

**Figure 7:**
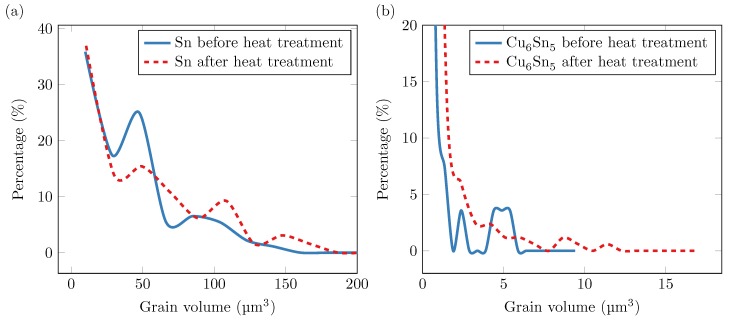
Histograms showing the grain size distribution before and after heat treatment: Sn grains (**a**); and Cu_6_Sn_5_ grains (**b**).

The sample initially contained 92 Sn grains. After the heat treatment, the tin grains coarsened so that only 65 of them remained. During the heat treatment, however, the 28 Cu_6_Sn_5_ grains in the original state increased to 170. The grain size distributions for Sn and Cu_6_Sn_5_ before and after the heat treatment are shown in Figure 7. In the original state, all Sn grains, except for the whisker, had a volume less than 140 $\mu^{3}$m. Assuming a cylindrical grain with length 7 μm, this corresponds to a grain diameter of 5.04 μm. The grain size distribution for the tin grains (Figure 7a) shows a large peak in the original state around 50 $\mu^{3}$m and a smaller peak around 100 $\mu^{3}$m. In the second measurement, both peaks shifted toward larger values due to grain growth. The peak at 5 $\mu^{3}$m in Figure 7b disappeared after the heat treatment. This can be explained by the formation of a large number of small grains of Cu_6_Sn_5_. The total volume of all grains of Cu_6_Sn_5_ increased from 38.3 $\mu^{3}$m to 231.5 $\mu^{3}$m during the heat treatment. Assuming that this volume of IMC would form a uniform layer in the sample, that layer would have grown from 78 nm to 472 nm. Using these numbers in Equation (1) with t=3 h and n=0.5 gives $A=227\;nm/\sqrt{h}$, which agrees well with the results presented by Deng et al. [11]. This suggests that the spatial resolution for the scanning 3DXRD measurements is high enough to give representative results regarding the growth of IMC.

### 3.2. Grain Boundary Misorientation

**Figure 8:**
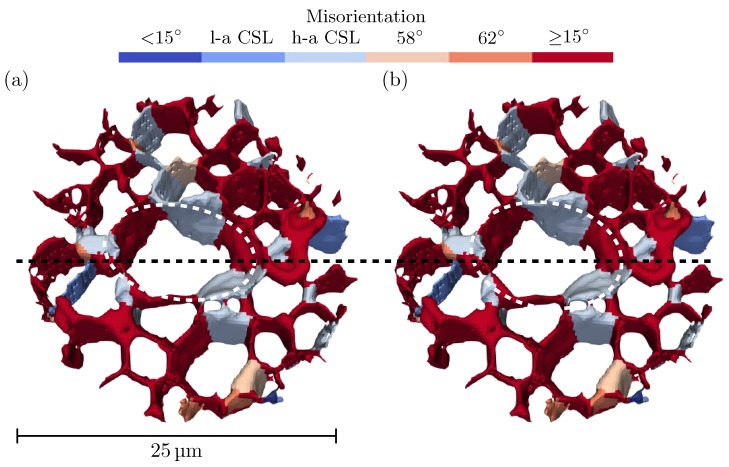
3D renderings of the grain boundary network before and after heat treatment. The grain boundaries are coloured based on their misorientation angle. Blue and light blue represent low-angle CSL (l-a CSL) and high-angle CSL (h-a CSL) boundaries, respectively. The dashed ellipses indicate the location of the whisker. The dashed black line indicates where the volume in Figure 4 is sliced: (**a**) before heat treatment; and (**b**) after heat treatment.

Figure 8 shows 3D renderings of the grain boundary network before and after heat treatment. The colours represent the misorientation angle of each grain boundary. Dark blue represents low-angle grain boundaries, i.e., grain boundaries with a misorientation less than 15${}^{\circ }$. Blue and light blue represent low-angle coincidence site lattice (CSL) boundaries and high-angle CSL boundaries, respectively. These are special misorientations with low grain boundary energy. For tin, the most common CSL boundaries are at misorientations of 7.3${}^{\circ }$, 14.3${}^{\circ }$, 22.3${}^{\circ }$, 28${}^{\circ }$, 43${}^{\circ }$, 58${}^{\circ }$, 62${}^{\circ }$, 71${}^{\circ }$, and 80${}^{\circ }$ [34]. The 58${}^{\circ }$ and 62${}^{\circ }$ misorientations are coloured in gray and orange, respectively. These angles correspond to twinning angles for twins on {1 0 1} and {3 0 1} planes, respectively [35]. High-angle grain boundaries with misorientations larger than 15${}^{\circ }$, but not within 1${}^{\circ }$ of any of the special misorientations, are coloured red. In Figure 8, it can be concluded that the majority of the grain boundaries are high-angle grain boundaries. Many of the grain boundaries have one of the special misorientations (either CSL or twin) with low grain boundary energy. All of the CSL boundaries except two are high-angle boundaries. In particular, it is noted that the whisker grain has high-angle CSL boundaries to three of the surrounding grains. It is also seen that all of the special grain boundaries (except two very short ones) seen in the first measurement were present even after heat treatment. This means that none of the grains with CSL boundaries was consumed during the grain growth.

**Figure 9:**
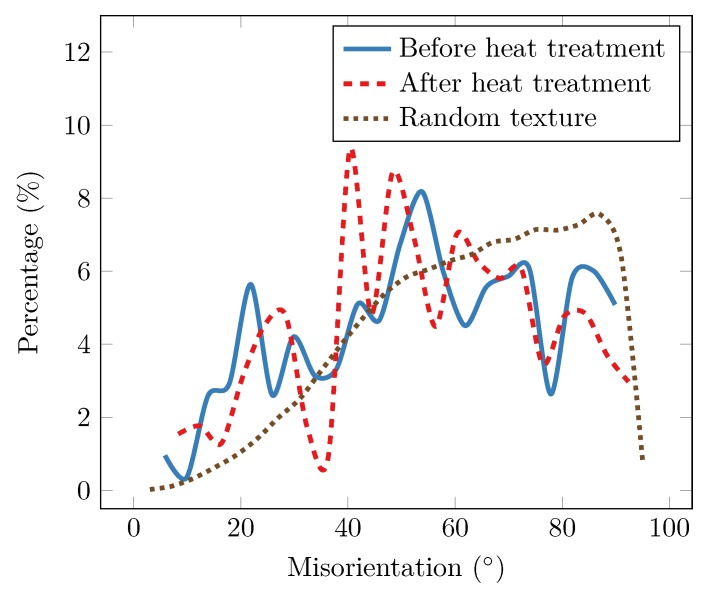
Histograms of the grain boundary misorientation before and after heat treatment. The bin size is 4${}^{\circ }$.

The distributions of misorientation between tin grains are plotted in Figure 9. The brown line shows the distribution for an assembly of randomly oriented tetragonal grains, taking the tetragonal symmetries into account the largest possible misorientation is 98.4${}^{\circ }$ [36,37]. In the measured distributions, misorientation angles larger than 60${}^{\circ }$ are less probable than in the random texture. Misorientations smaller than 30${}^{\circ }$ are, on the other hand, more common than in the random texture. In the original state, the most common misorientation was between 52° and 56${}^{\circ }$. After heat treatment this misorientation peak split into two peaks, one located between 48° and 52${}^{\circ }$, and a smaller peak between 58° and 62${}^{\circ }$. The formation of a peak between 58° and 62${}^{\circ }$, which are twin misorientation angles, indicates that, during the heat treatment, grains reoriented to configurations with lower grain boundary energy.

### 3.3. Growth and Crystal Structure of Cu_6_Sn_5_

**Figure 10:**
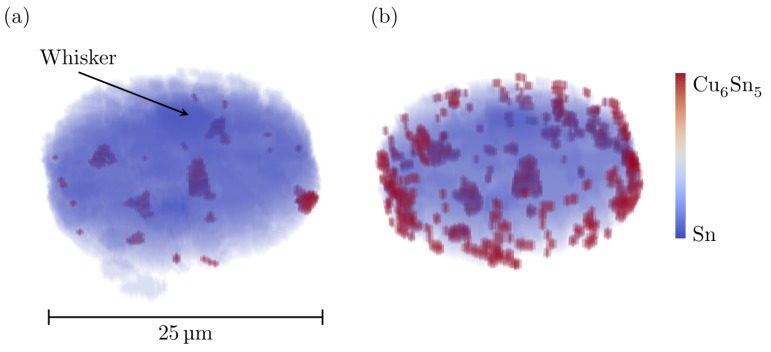
3D map showing the location of Cu_6_Sn_5_ grains (red) in the Sn coating (blue): (**a**) before heat treatment; and (**b**) after heat treatment of 150 °C for three hours.

Figure 10 shows 3D maps of the positions of the Cu_6_Sn_5_ grains within the tin coating, before and after the heat treatment. No continuous layer of IMC can be identified; instead, discrete grains can be seen located in the deeper part of the tin coating, close to the copper substrate. Since the IMC binds the tin coating and the copper substrate together, it is expected that there is a continuous layer on the nanometre scale, too small to see in these measurements. Evidently, the amount of the intermetallic phase increased during the heat treatment. Most of the new IMC growth or formation took place along the edges of the sample. This is most likely due to the presence of redeposited, amorphous material from the FIB milling. Apart from the IMC at the edge of the sample, most IMC grains formed at the bottom of the tin layer rather than as grain boundary precipitates. The largest IMC grain before the heat treatment was located just below the whisker. The presence of large IMC particles below the whisker has previously been reported [20,32,38]. This suggests that large IMC grains can act as preferred nucleation sites for tin whiskers. This might be related to high compressive stresses building up due to the formation of IMC, which is then relaxed by the nucleation and growth of the whisker.

**Figure 11:**
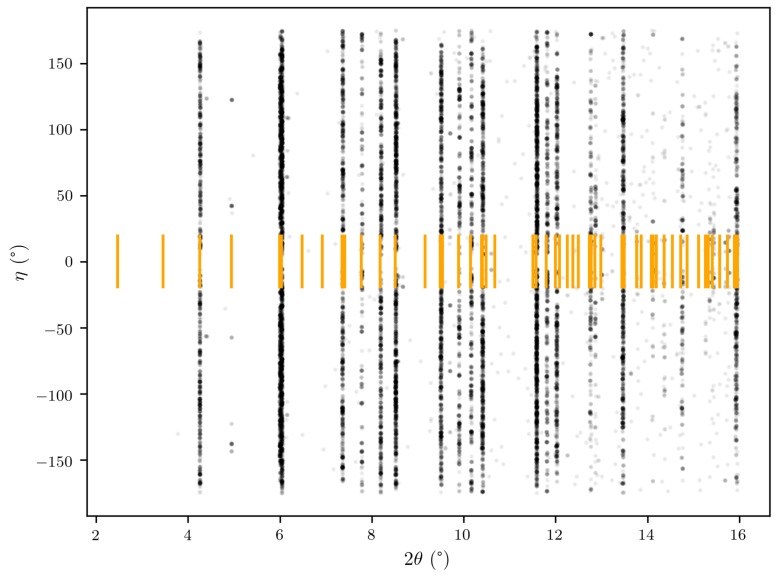
Indexation of peaks not belonging to tin grains for one horizontal slice through the heat treated sample. The position of each diffraction peak is given by the 2θ and η angles (cf. Figure 2). The orange lines indicate the theoretical 2θ angle of diffraction peaks originating from a powder of hexagonal Cu_6_Sn_5_.

In the Cu–Sn phase diagram, there is a polymorphic transformation from the high temperature hexagonal *η*–Cu_6_Sn_5_ to a monoclinic $\eta^{\prime }$–Cu_6_Sn_5_ occurring at 186 ${}^{\circ }C$ [39]. Although the experiment took place at temperatures lower than 186 ${}^{\circ }C$, the IMC in the sample was indexed by the hexagonal η–Cu_6_Sn_5_ phase. Figure 11 shows the (2θ,η) position (cf. Figure 2) of all peaks not belonging to any tin grain in one of the horizontal slices through the 3D image of the heat-treated sample. The orange lines indicate the theoretical peak positions from a powder of hexagonal Cu_6_Sn_5_. It is clear that the theoretical and measured peak positions match. The absence of superstructure peaks and the lack of monoclinic distortion in the indexed Cu_6_Sn_5_ grains supports the choice of modelling the IMC as hexagonal. An explanation for the presence of hexagonal IMC could be that, during sample preparation, either during deposition or FIB milling, the temperature was locally higher than the transformation temperature. The cooling rate was then too high for the η → $\eta^{\prime }$ transformation to complete and the *η* phase was left in a metastable state [40,41,42]. The IMC seen after the heat treatment was also indexed as a hexagonal phase, indicating that it is probably not a formation of new crystals but rather the growth of grains already present from the sample preparation, too small to see in the measurement before heat treatment.

### 3.4. Stress Field

**Figure 12:**
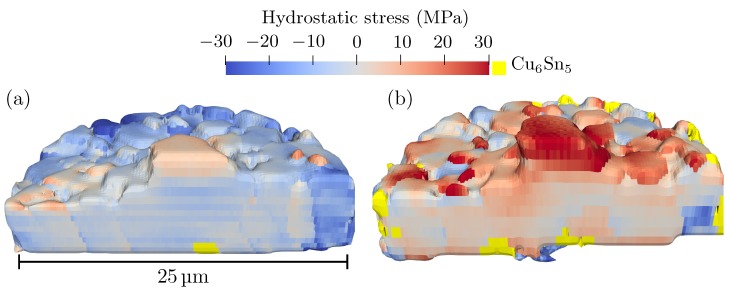
3D reconstruction of hydrostatic stress field in the tin coating; the volume is sliced to reveal the internal structure. The stresses are calculated using the mean lattice parameters of the whisker as a stress-free reference. Yellow colour indicates Cu_6_Sn_5_: (**a**) before heat treatment; and (**b**) after heat treatment of 150 ${}^{\circ }C$ for three hours.

The 3D maps in Figure 12 are coloured by the hydrostatic stress field in the tin coating; blue indicates compression and red indicates tension whilst the IMC grains are coloured yellow. The intragranular variations seen in the stress fields would not be possible to capture using standard 3DXRD (using a box or line beam), which proves the usefulness of the scanning based approach taken in this work. The stresses are calculated using the elastic constants given in Table 2 and the following lattice parameters were taken as a stress-free reference, a=b= 5.812 Å, c= 3.170 Å, α=β=γ= 90${}^{\circ }$. These correspond to the average lattice parameters of the whisker in the original state. It must be emphasised that these lattice parameters do not necessarily correspond to the true stress-free state of the material. The level of stress can, therefore, only be compared in a relative way.

In the original state, the level of hydrostatic stress in the coating was relatively low. It is also seen that the hydrostatic stress was mainly compressive, the few regions with tensile stress were all located at the surface or along grain boundaries, in particular, the part of the whisker above the surface has tensile stress. After heat treatment, the hydrostatic stress field was far less homogeneous than in the original state. In some regions, the hydrostatic stress became tensile, while for other regions the stress remained compressive. It is interesting to note that, despite a large amount of IMC formation, which is known to introduce compressive stress in tin coatings, the stress after heat treatment was less compressive than before. This evolution of the stress field indicates that grain growth that occurred during the heat treatment also caused stress relaxation in the coating. Regions of compressive stress were, however, present close to many of the grains of Cu_6_Sn_5_. Finally, it is noted that the stress in the whisker became more tensile after the heat treatment. This was unexpected since the part of the whisker above the surface should be a defect-free single crystal and, therefore, be stress-free [44]. A possible explanation is that the tensile stresses was caused by the mismatch in thermal expansion between the tin and platinum, which was redeposited on the whisker during the FIB milling. This redeposition should be a local effect that does not affect the stress field in the rest of the coating. In Figure 1, it is seen that there was some redeposition at the top of the whisker.

In order for the whisker to grow, it is necessary to have diffusion of material to its root. The diffusion is believed to be driven by negative gradients in hydrostatic stress, such that material flows from more compressed to less compressed regions. Sobiech et al. [7] found a negative, in-plane, strain gradient when averaging radially from the root of the whisker. In a more recent study, no average gradient was observed; instead, gradients were found locally in specific directions that changed with ageing time [10]. Both studies used Laue diffraction, where the hydrostatic part of the strain tensor could only be approximated using some assumption, e.g., that $\sigma_{zz}=0$. The stress fields in Figure 12 are the first reconstructions of the hydrostatic stress in the vicinity of a tin whisker not based on any assumptions regarding the stress state.

**Figure 13:**
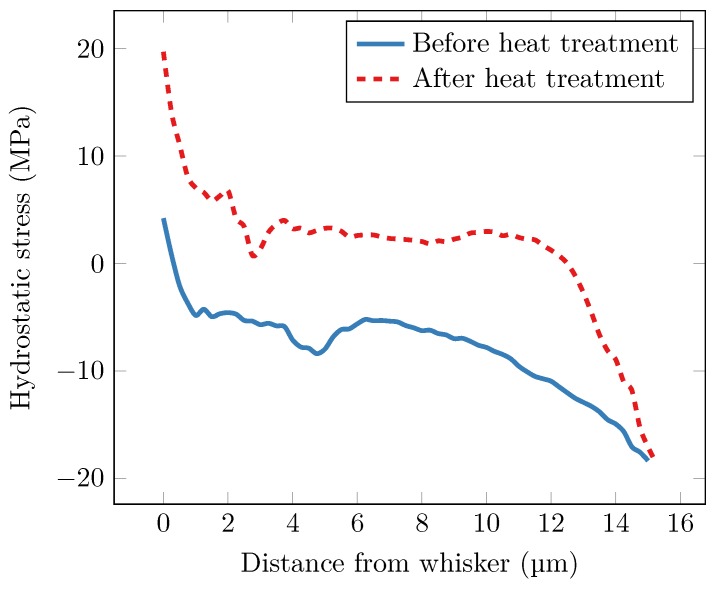
Hydrostatic stress as a function of distance from the whisker. Distance 0 is taken in the centre of the whisker grain, at level with the sample surface.

Figure 13 shows the hydrostatic stress, radially averaged in 3D. Distance zero was taken in the centre of the whisker grain level with the sample surface; the part of the whisker above the surface was not included in the averaging. In the initial state, the stress close to the whisker root was almost zero. Increasingly compressive stress can be seen until approximately 4 μm from the whisker, where the stress gradient changes direction and the stress becomes less compressive. From 6 to 15 μm, the stress becomes increasingly compressive again. The initial stress gradient would drive diffusion of tin atoms towards the whisker root, from at least 4 μm, which is also the length of the strain gradient observed in Sobiech et al. [7]. Even though the studied whisker was relatively small (approximately 10 μm long and 5 μm in diameter), it seems unlikely that all material making up the whisker originated from within 4 μm of the root. Radial diffusion of atoms towards the root was, therefore, probably not the only driving force for tin whisker growth. Instead, the supply of material likely came from a combination of long-range gradients in specific directions (such as those observed in Hektor et al. [10]) and the radial gradient found here.

After heat treatment, the hydrostatic stress was more tensile. A negative gradient can again be seen for the first few micrometres. After that, the curve flattens out before the stress becomes increasingly compressive again. It should be noted that the stress field close to the edge of the sample was likely affected by the boundaries of the sample. The steep gradient seen close to the edge of the sample, from 12 to 15 μm, might have been caused by the large amount of IMC formed from redeposited material on the sample walls. It could also have been affected by the sample preparation procedure, e.g., by the deposition of Pt to prevent the edges from deforming during milling. The influence of some of these effects could have been minimised by making the sample larger, which would, however, have either increased the measurement time or decreased the spatial resolution.

## 4. Conclusions

Scanning 3DXRD was used to study the microstructure around a tin whisker with a spatial resolution of 250 nm. The effect of heat treatment on the sample was also investigated. It was found that the deposited Sn formed a columnar grain structure with a [1 0 0] fibre texture such that the crystals *c*-axes were parallel to the copper substrate. During heat treatment, grains with orientations far from [1 0 0] or [1 1 0] disappeared or reoriented to match with the texture; the heat treatment also appeared to have facilitated the growth of IMC. Most of the new IMC was located along the edges of the sample, likely due to redeposition of Cu during sample preparation. The new IMC in the bulk of the sample formed close to the interface between the copper substrate and the tin coating. The largest IMC grain was located just below the whisker, suggesting that large IMC grains act as nucleation sites for whiskers. The Cu_6_Sn_5_ phase was well indexed using the hexagonal unit cell of η-Cu_6_Sn_5_, which is stable at temperatures above 186 ${}^{\circ }C$. This suggests that the η-phase can exist in a metastable state for a long time without transforming to the monoclinic $\eta^{\prime }$ stable at room temperature.

The hydrostatic stress field around the whisker could be reconstructed in 3D. It was found that, in the initial state, the stress in the whisker was less compressive than in the rest of the coating. After heat treatment, the stress became more tensile in some regions while remaining compressive in others. The compressive stress was mainly located close to grains of Cu_6_Sn_5_. A radially averaged negative stress gradient was found, extending for approximately 4 μm from the whisker root. This gradient, together with more extended gradients in specific directions (observed in [10]), is believed to have provided the driving force for diffusion of material to the root of the whisker.

## Figures and Tables

**Table 1:** Reference lattice parameters and crystal structure used for indexing of the measured diffraction patterns.

Phase	Structure	Lengths (Å)			Angles (${}^{\circ }$)		
		a	b	c	α	β	γ
Sn	Body-centered tetragonal	5.811	5.811	3.173	90	90	90
Cu_6_Sn_5_	Hexagonal	4.186	4.186	5.074	90	90	120

**Table 2:** Elastic constants (GPa) for tin [43].

$C_{11}$	$C_{22}$	$C_{33}$	$C_{44}$	$C_{55}$	$C_{66}$	$C_{12}$	$C_{13}$	$C_{23}$
72.3	72.3	88.4	22.0	22.0	24.0	59.4	35.8	35.8
